# Databases Selection in a Systematic Review of the Association between Anthropometric Measurements and Dental Caries among Children in Asia

**DOI:** 10.3390/children8070565

**Published:** 2021-06-30

**Authors:** Rokiah Mamikutty, Ameera Syafiqah Aly, Jamaludin Marhazlinda

**Affiliations:** 1 Department of Community Oral Health and Clinical Prevention, Faculty of Dentistry, University of Malaya, Kuala Lumpur 50603, Malaysia; rokiah73@gmail.com (R.M.); drfasya@gmail.com (A.S.A.); 2 Oral Health Programme, Ministry of Health Malaysia, Level 5, Block E10, Parcel E, Precinct 1, Federal Government Administrative Centre, Putrajaya 62590, Malaysia

**Keywords:** databases, Asia, dental caries, anthropometric measurements, systematic review

## Abstract

A comprehensive search for primary studies using a sufficient number and relevant databases is critical to minimise bias and increase the validity of a systematic review. We examined the frequency and choices of databases commonly used to provide an efficient search of primary studies for a systematic review of anthropometric measurements and dental caries among children in Asia. Twelve previous systematic reviews on a similar topic were retrieved from six databases. The frequency and choice of databases used by reviewers were determined from the methods sections. We also identified the lists of other databases usually searched in other reviews. Eligibility criteria for final databases selection were the database’s scope, the topic of interest, design of the study, type of article, and the accessibility of the databases. Of the 77 databases identified, previous reviews on this topic used 21 databases, ranging from 2 to 12 databases in each review. Medline, Cochrane Library, Web of Science, and PubMed were employed most frequently. Twenty-six databases were eligible and selected for the present review. Twelve were regional databases to provide comprehensive coverage of primary studies. A systematic approach in selecting appropriate databases for searching primary studies is paramount to reduce errors, ensure coverage, and increase the validity of systematic reviews’ conclusions.

## 1. Introduction

Searching primary studies is deemed as one of the critical steps in performing systematic review and meta-analysis. Failure to comprehensively search for primary studies for a systematic review may incur biased results. Importantly, databases are the primary source for searching potential studies; thus, the choice of databases may affect search quality [1]. Hence, systematic planning in selecting appropriate databases is mandatory.

Several databases are available for searching primary studies, and the number is growing. These electronic databases can be divided into the bibliographic database, subject-specific database, regional database, grey literature database, clinical trial registries, and web search [2]. Medline, EMBASE, and the Cochrane Central Register of Controlled Trials (CENTRAL) are essential bibliographic databases. Medline covers 25 million references that indexes with Medical Subject Heading (MeSH). While EMBASE includes important biomedicine references from 90 countries, it covers all Medline records and more, available via subscription. Each database has its purpose; for instance, CENTRAL is an important source for randomized trials. The subject-specific database CINAHL (nursing and allied health) is a desired database for nursing-related topics, and the ProQuest Dissertations and Theses Database searches for theses and dissertations related to the review.

There are various opinions about the number of databases deemed sufficient for searching primary studies in a systematic review. Vassar et al. (2017) recommended using at least two databases for a review [1]. Referring to the commonly used databases such as EMBASE, Medline, Web of Science, and Google Scholar, Bramer et al. (2017) suggested that a systematic review requires four databases to ensure adequate coverage of the review topic [3]. Likewise, Higgins et al. (2019) pointed out that searching multiple databases may provide more excellent coverage than searching a single database [2]. Moreover, the overlapping of search results is less, and it also minimized selection bias.

Selecting databases should also consider the review topic [4,5]. In some instances, the reviewer might need to identify and search for additional databases [2,6]. For example, the present review includes primary studies done in Asian countries. Consequently, regional databases that focus on the literature produced in the Asian regions need to be considered. More than a few regional or local databases of Asian countries are available, for example, IndMED, KoreaMed, and Chinese Biomedical Literature Database. These databases provide useful information, especially regarding studies published in Asian regions. However, regional databases may have some limitations. Saokaew et al. (2015) noticed that some databases from Japan and Thailand had limited accessibility [7]. In the view of the scarcity of resources, accessibility issues in some databases may limit a researcher to include the databases for searching for primary studies.

On the other hand, even though selecting multiple databases provides better coverage, time and resources could serve as the reviewers’ limitations [2]. Using numerous databases is very time-consuming. Furthermore, the syntax of search strategies is database-specific [3]. The number of retrieved articles will be enormous; therefore, screening and excluding the irrelevant studies may jeopardize the time required to complete a systematic review [4]. Hence, a balanced decision is needed. Accordingly, to achieve a balanced decision, the selection of databases should be made systematically and understanding the purpose of additional databases is required. Little evidence was found to guide such a process, especially concerning the number of electronic databases and the choice of databases should be searched [8]. Specific criteria were discussed, which consisted of, (i) the inclusion of the core databases such as Medline, EMBASE, Cochrane Library, Web of Science, and Google Scholar [2,3], (ii) the inclusion of additional databases guided by the review topic [4], and (iii) the inclusion of regional databases due appropriate [2]. The reviewer should also consider the resources and accessibility of the databases in the final selection of databases. The present review investigated the association between anthropometric measurements and dental caries in children in Asia. Thus, this paper aimed to identify commonly used and most relevant databases to provide an efficient search of primary studies for this review.

## 2. Materials and Methods

The researcher performed a search for systematic reviews and meta-analyses of anthropometric measurements and dental caries in children from the following six databases: Medline, PubMed, Web of Science, Scopus, CINAHL, and Google Scholar. The search was performed from the inception of the databases to 30 June 2020. The search strategies built by the information specialist for the present review on ‘Anthropometric measurements and dental caries among children in Asia’ was adapted. The six databases we used and the search strategies for PubMed in Table 1 is an example for adaption. Due to the ability to explode narrower terms, PubMed was chosen as a primary database to build the search strategy, and was then adapted to the other five databases. After that, two trained and calibrated researchers (Kappa score for title and abstract screening = 0.96, *p* < 0.05; full-text screening = 0.85, *p* < 0.05) independently screened the retrieved review articles at two levels, the title and abstract, and the full text of the remaining systematic reviews and meta-analyses. Of the selected systematic reviews and meta-analyses, the first researcher extracted data on the databases reported in each study’s method section, number and category of databases used, and number of articles retrieved in each database. Upon completion of this process, the data extracted was validated by the second researcher. Besides, the researcher also identified and listed all the commonly used databases for systematic review studies [9], including the databases suggested by articles, books, website, and courses.

**Figure 1:**
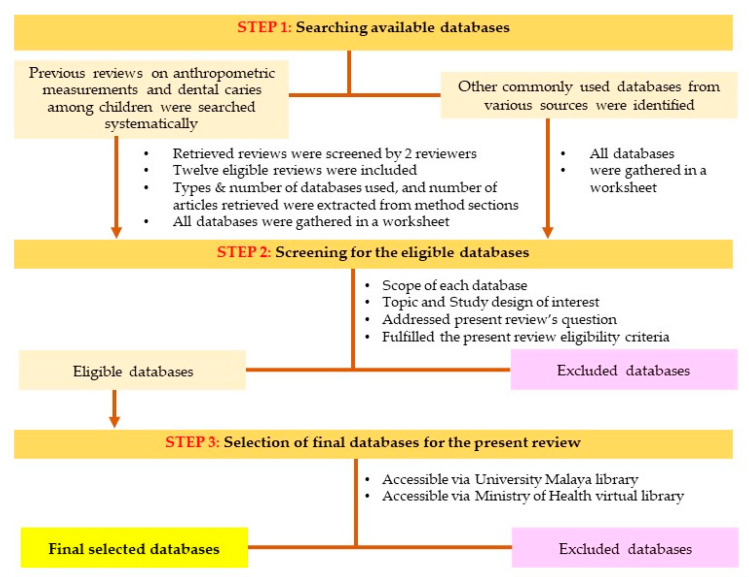
Flowchart for the process of databases selection for the present review.

Subsequently, the scope of each database such as the topics or areas of interest, design of study, and types of articles accepted were recorded. Of all the previously identified databases, the eligible databases for searching primary studies addressing the present review question and its eligibility criteria were selected. The inclusion and exclusion criteria of the present review are shown in Table 2. As such, databases for grey literatures, review papers, intervention studies, and regional databases that do not include studies in Asia were removed. Reasons for the exclusion of any database were recorded. Of those eligible databases, only those could be accessed via either the University Malaya Library (UM Library) or the virtual library of the Ministry of Health Malaysia (MOH) formed the final databases included for the present review. A Microsoft Excel spreadsheet was used to tabulate information describing each database including the database category, the name and purpose of the databases, their accessibility, frequency used, charges/fees if applicable, and the URL of the databases. The processes of selecting the final databases for the present review are summarized in Figure 1.

## 3. Results

**Figure 2:**
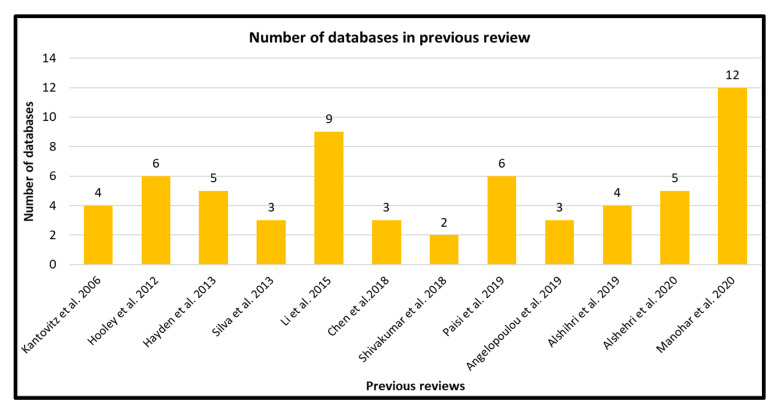
Number of databases used in previous systematic reviews.

Twelve systematic reviews and meta-analyses on anthropometric measurements and dental caries in children were retrieved [10,11,12,13,14,15,16,17,18,19,20,21]. The number of databases used in each systematic review ranged from 2 to 12 (Figure 2).

**Figure 3:**
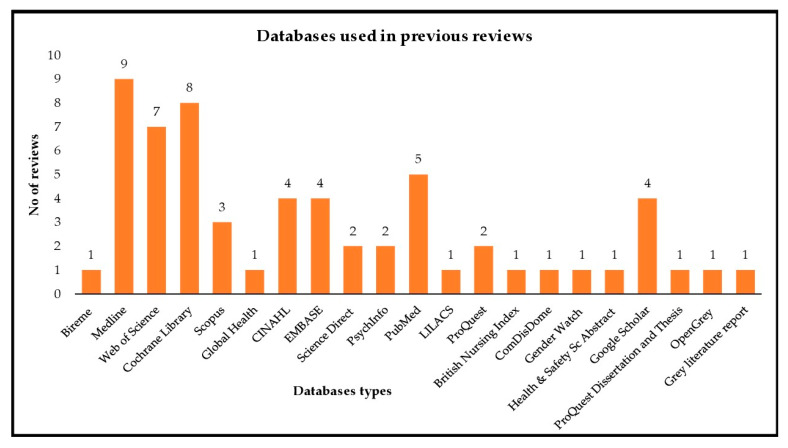
Number of databases used in previous systematic reviews.

**Figure 4:**
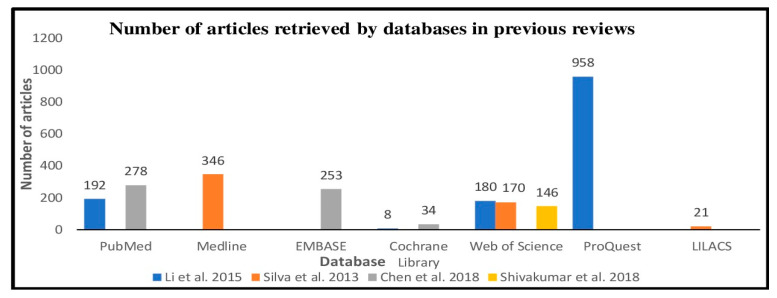
Number of articles retrieved by each database in previous reviews.

A total of 21 databases were used in searching primary studies for the review topic (Figure 3). The most frequently used databases were Medline in nine reviews, Cochrane Library in eight reviews, Web of Science used in seven reviews and, PubMed in five reviews. The review that mentioned using PubMed/Medline was grouped under Medline. Among these databases, ProQuest was found to recall the greatest number of articles, followed by Medline, PubMed, Embase, Web of Science, LILACS, and Cochrane the least (Figure 4). However, these results were derived from four reviews, i.e., Silva et al., 2013, Li et al., 2015, Chen et al., 2018, and Shivakumar et al., 2018, which provided information regarding the number of studies retrieved by each database.

Considering the databases discovered from the 12 reviews on this topic, databases commonly used for systematic reviews (*n* = 21), and other sources, a total of 77 databases divided into 8 categories, namely, bibliographic database (*n* = 5), subject-specific database (*n* = 3), regional database (*n* = 24), grey literature database (*n* = 6), clinical trial registries (*n* = 1), web search (*n* = 1), citation indexes (*n* = 2), and others (*n* = 35) were identified (Table 3).

**Figure 5:**
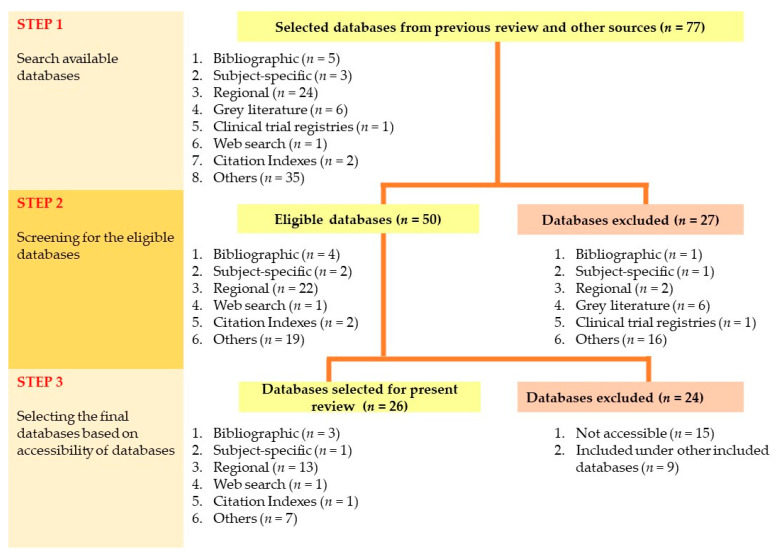
Summary of results at each stage of selecting databases for the present review.

After checking against the scope of each database, 27 databases (Table 4) were removed due to irrelevancy of study design and topic of interest of the databases to the present review, leaving only 50 eligible databases (Table 5). The list of these databases with reasons for their exclusion is presented in Table 4. Of the eligible databases, 15 databases were excluded as they were not accessible through the University Malaya Library (UM Library) or the virtual library of the Ministry of Health Malaysia, and nine other databases were also excluded as they were subset databases or already part of other collection databases such as ProQuest, Global Index Medicus, EBSCOhost, and Web of Science (Table 6). As such, only a total of 26 remaining databases were finally included in the present review which include Medline (Ovid), Pubmed, Scopus, Web of Science, Cochrane Library, CINAHL, LILACS, Science Direct (EBSCOhost), Dentistry and Oral Science Source (EBSCOhost), KoreaMed, Thai Index Medicus, Thai Medical Index, Bibliography of Asian Studies, HERDIN, Psychology and Behaviour Science (EBSCOhost), IDRnet, EMRpub, Global Index Medicus (include IMEMR, IMSEAR, WPRIM), CKNI, SaudiMedLit, Hong Kong Literature Database, Cinii, ProQuest, Google Scholar, Scopus, Cochrane Library, E-journal and SocINDEX and Health Business Elite (EBSCOhost) and TRIP (Table 6). The reasons for excluding other databases are displayed in Table 7. The results at each stage for selecting the final databases for the present review are summarized in Figure 5.

## 4. Discussion

This paper aimed to identify all appropriate databases for the present systematic review and meta-analysis regarding the ‘Association between anthropometric measurements and dental caries among children in Asia’. There were limited articles and guidance regarding a systematic approach in choosing databases for a systematic review [8]. Thus, the steps used in this study were developed based on a few related sources [1,2,3,8]. Of the 77 identified databases, only a final of 26 databases were eligible to provide the most relevant search for primary studies for our review. Three of the four primary databases suggested by Bramer et al. (2017) had been selected, namely, Medline, Web of Science and Google Scholar [3].

EMBASE was not chosen as this database is only accessible via a paid subscription and is not covered either under UM Library or the Ministry of Health Malaysia’s virtual library. EMBASE is generally considered one of the key international databases for general healthcare and has an acceptable recall of primary studies in a systematic review [3]. One of EMBASE’s strengths, is that it includes articles from about 90 countries and provides access to almost 2 million records [2]. Simultaneously, if MEDLINE, Web of Science, and Google Scholar were combined with Cochrane CENTRAL, a similar recall of primary studies could be achieved [3,22]. Furthermore, Scopus was also found to have the capability to retrieve 100% of all included references retrieved by either EMBASE or Web of Science in some reviews [3]. Hence, we anticipated that adding Scopus, Web of Science, and Cochrane Library databases for our review would negate the limitation that we faced by not including EMBASE.

In agreement with Bramer et al. (2017), our findings from 12 systematic reviews [10,11,12,13,14,15,16,17,18] also showed that the most frequently-used databases were Medline, PubMed, Cochrane Library, and Web of Science [3]. Another two databases, i.e., PsycINFO and Global Health databases, are inaccessible via UM Library or the virtual library service by the Ministry of Health Malaysia and were thus not selected for the present review. Nonetheless, PsycINFO only retrieved a small percentage of added unique references [3], hence it is fair not including this database. Moreover, we decided to complement our search with regional databases, especially those from Asian countries, to complete our quest.

From the previous reviews on anthropometric measurements and dental caries, the minimum number of databases used was 2 and the maximum number was 12. The vast difference in the number of databases selected in our review (26 databases) compared to previous systematic reviews is because our review collects evidence from Asian countries. Hence, we included several regional databases to complement studies not retrieved in the major databases. There is no specific guidance in terms of the appropriate number of databases to provide a recall of primary studies efficiently [3]. Vassar et al. (2017), suggested that using additional databases, as opposed to core databases, might provide better coverage of primary studies [1]. While Rathbone et al. (2016) revealed that coverage of different databases may vary due to the scope and context of each database, multiple databases search is thus beneficial [23].

On the contrary, Hartling et al. (2016), revealed that most of the relevant studies could be acceptably found within a limited number of databases and therefore suggested that selective searching might not introduce bias in terms of effect estimates [8]. Other factors such as building the sensitive search strategies [24] and choosing databases that are the most relevant to the topic at hand may play roles in balancing up the quantity of databases and the quality of search [25]. This could explain the contradictory findings by Hartling et al. (2016) [8] and Vassar et al. (2017) [1].

Accordingly, the findings from the previous 12 systematic reviews showed that the number of articles retrieved from various databases was associated with the review topic [5]. For instance, the topic of association between anthropometric measurements and dental caries mostly involves observational studies. Consequently, the Cochrane Library, a database for RCT studies, returned the least number of primary studies in the previous systematic review compared to other databases. Noticing various types of databases, we retrieved different kinds of articles, selecting the eligible databases that were made by the scope of databases, study design, review’s topic, and eligibility criteria of the present review. Some databases are specific to types of study design and regions of the articles. For example, DARE, the Campbell Library, Clinical Trial Registries are specific to review articles and trials that were not eligible for the present review. On the other hand, databases such as IMSEAR provide additional articles specific to the Southeast Asia region and would benefit the review. Hence, a decision on selecting databases should be based on knowing and understanding the nature of the databases. However, not all eligible databases are accessible to researchers. As such, final selection would be restricted to resources and accessibility of the databases.

## 5. Conclusions

The selection of databases depends on a few factors such as the topic of the review, the study design included in the review, resources, and database accessibility. It is also important to note that a range of regional databases was also included in the current review. After a thorough selection, the present review will be performing primary studies searching using the 26 selected databases.

## Figures and Tables

**Table 1:** PubMed, with its ability to explode narrower terms, built the search strategies of the three following concepts ‘children’, ‘anthropometric measurements’, and ‘caries’, then adapted it to other five databases.

Search Strategy for PubMed		Results
#1	“child” [MeSH Terms] OR “child” [All Fields] OR “children” [All Fields] OR “child s” [All Fields] OR “children s” [All Fields] OR “childrens” [All Fields] OR “childs” [All Fields] OR “adolescences” [All Fields] OR “adolescency” [All Fields] OR “adolescent” [MeSH Terms] OR “adolescent” [All Fields] OR “adolescence” [All Fields] OR “adolescents” [All Fields] OR “adolescent s” [All Fields] OR “toddler” [All Fields] OR “toddler s” [All Fields] OR “toddlers” [All Fields] OR “adolescent” [MeSH Terms] OR “adolescent” [All Fields] OR “teen” [All Fields] OR “adolescent” [MeSH Terms] OR “adolescent” [All Fields] OR “youth” [All Fields] OR “youths” [All Fields] OR “youth s” [All Fields]	3,784,836
#2	“anthropometry” [MeSH Terms] OR “body fat distribution” [MeSH Terms] OR “waist hip ratio” [MeSH Terms] OR “waist height ratio” [MeSH Terms] OR “skinfold thickness” [MeSH Terms] OR “waist circumference” [MeSH Terms] OR “obesity” [MeSH Terms] OR “body mass index” [All Fields] OR (“waist height ratio” [MeSH Terms] OR (“waist height” [All Fields] AND “ratio” [All Fields]) OR “waist height ratio” [All Fields] OR (“waist” [All Fields] AND “height” [All Fields] AND “ratio” [All Fields]) OR “waist height ratio” [All Fields]) OR (“skinfold thickness” [MeSH Terms] OR (“skinfold” [All Fields] AND “thickness” [All Fields]) OR “skinfold thickness” [All Fields]) OR (“waist hip ratio” [MeSH Terms] OR (“waist hip” [All Fields] AND “ratio” [All Fields]) OR “waist hip ratio” [All Fields] OR (“waist” [All Fields] AND “hip” [All Fields] AND “ratio” [All Fields]) OR “waist hip ratio” [All Fields]) OR (“waist circumference” [MeSH Terms] OR (“waist” [All Fields] AND “circumference” [All Fields]) OR “waist circumference” [All Fields]) OR (“anthropometries” [All Fields] OR “anthropometry” [MeSH Terms] OR “anthropometry” [All Fields]) OR (“obeses” [All Fields] OR “obesity” [MeSH Terms] OR “obesity” [All Fields] OR “obese” [All Fields] OR “obesities” [All Fields] OR “obesity s” [All Fields])	871,247
#3	“caries” [All Fields] OR “dental caries” [MeSH Terms] OR (“dental” [All Fields] AND “caries” [All Fields]) OR “dental caries” [All Fields] OR “caries” [All Fields] OR (“dental caries” [MeSH Terms] OR (“dental” [All Fields] AND “caries” [All Fields]) OR “dental caries” [All Fields]) OR (“dental caries” [MeSH Terms] OR (“dental” [All Fields] AND “caries” [All Fields]) OR “dental caries” [All Fields] OR (“dental” [All Fields] AND “decay” [All Fields]) OR “dental decay” [All Fields]) OR (“dental caries” [MeSH Terms] OR (“dental” [All Fields] AND “caries” [All Fields]) OR “dental caries” [All Fields] OR (“tooth” [All Fields] AND “decay” [All Fields]) OR “tooth decay” [All Fields])	62,469
#4	**Search:** ((#1) AND (#2)) AND (#3) **Filters:** Meta-Analysis, Review, Systematic Review	91

Footnote: #1: search strategy for ‘children’ concept; #2: search strategy for ‘anthropometric measurements’ concept; #3: search strategy for ‘caries’ concept; #4: all the three strategies combined with Boolean operator ‘AND’ and limit by study design.

**Table 2:** Eligibility criteria of the present review.

		Inclusion	Exclusion
1	Type of studies	**Observational studies:** cross-sectional, comparative cross-sectional, case-control, nested case-control, retrospective prospective cohort study design	case series case report intervention/ experimental study
2	Type of population	**Children** age 19-year-old and below both genders in Asian countries	Studies of population restricted to a specific disease, condition, or metabolic disorders.
3	Type of exposure	**Anthropometric measurements** Body mass index (BMI) Waist circumference (WC) Waist-to-hip ratio (WHR) Waist-to-height ratio (WHtR) Skinfold thickness (SFT)	
4	Type of outcome	Association anthropometric measurement and dental caries	Exclude if it does not examine the association of anthropometric measurements with dental caries
5	Others	Full text published article in English language	Thesis, dissertations

**Table 3:** List of all databases found from the literature review (*n* = 77).

No	Database Category	Databases
1	Bibliographic database	1. Cochrane library 2. Database of Abstract of Review of Effect (DARE) 3. Embase (biomedical, with an emphasis on drugs and pharmaceuticals, more non-US coverage than MEDLINE 4. MEDLINE 5. PubMed (biology and health science)
2	Subject-specific	1. POPLINE (population, family planning and reproductive) 2. Cumulative Index to Nursing and Allied Health Literature (CINAHL) 3. PsycINFO
3	Regional databases	1. African Index Medicus (AIM) 2. Latin American and Caribbean Health Science literature (LILACS) 3. China: Chinese biomedical literature Database (CBM) 4. India: IndMED 5. Korea: KoreaMed 6. South-east Asia: Index Medicus for the South-East Asia Region (IMSEAR) 7. China National Knowledge Infrastructure (CNKI) 8. Chinese Scientific Journal Database (VIP) 9. Russian Medical and Health Journals (UDB-MED) 10. SaudiMedLit 11. Thai Index Medicus 12. Thai Journal Citation Index Centre 13. Thai Medical Index 14. Western Pacific Region Index Medicus (WPRIM) 15. WAN FANG 16. South Asian Database of Controlled Clinical Trials 17. Japan: Nikkei Asian Review 18. Korea: RISS- Korean Education and Research Information Service 19. Hong Kong: Hong Kong Literature Database 20. Asia: Bibliography of Asian Studies 21. Health Research and Development Information Network (HERDIN) 22. Japan: Cinii 23. INRnet 24. EMRpub
4	Grey literature database	1. New York Academy of Medicine Grey Literature Report 2. QAIster 3. ProQuest Dissertation and Theses Database (PQDT) 4. System for information on Grey literature in Europe (OpenSIGLE) 5. OpenGrey 6. Grey literature report
5	Clinical trial registries	1. Clinical trial registries
6	Web search	1. Google Scholar (first 200 references as sorted in the relevance ranking [3])
7	Citation Indexes	1. Science Citation Index 2. Scopus
8	Others	1. The Campbell Library (systematic review and protocol) 2. Web of Science 3. SciELO 4. ERIC (Education) 5. IBIDS (international bibliographic information on dietary supplements) 6. TRIP 7. ScienceDirect 8. BNI (British Nursing Index) 9. ICL (index of chiropractic literature) 10. NAPS (new abstract and paper in sleep) 11. OT seeker (occupational therapy systematic evaluation of evidence) 12. PEDRO (physiotherapy evidence database) 13. PILOTS (PTSD and traumatic stress) 14. RDRB (research and development resource base- medical education) 15. RehabData (rehabilitation) 16. Social Care Online (social work and mental health) 17. TOXNET (toxicology) 18. TRIS (Transportation Research Information Service) 19. ChildData (child-related topic) 20. EMcare (nursing and allied health) 21. CommunityWISE (community issue) 22. HaPI (Health and Psychosocial Instruments) 23. IPA (International Pharmaceutical Abstracts) 24. MANTIS (Manual Alternative and Natural Therapy Index System) 25. Sociological abstracts 26. Global Health 27. Dentistry and Oral Science Source via EBSCOhost 28. Psychology and Behaviour Science via EBSCOhost 29. E-journal and SocINDEX and Health Business Elite via EBSCOhost 30. Global Index Medicus 31. ProQuest 32. Joanna Briggs Institute EBP Database 33. ComDisDome 34. Gender Watch 35. Health and Safety Science abstract

**Table 4:** List of databases excluded following the review title and scope (*n* = 27).

		Database	Reason to Exclude
1	Bibliographic database	1. Database of Abstract of Review of Effect (DARE)	# for review
2	Subject-specific	1. POPLINE (population, family planning and reproductive)	# not relevant
3	Reginal databases	1. African Index Medicus (AIM)	# not relevant
		2. South Asian Database of Controlled Clinical Trials	# for trials
4	Grey literature database	1. New York Academy of Medicine Grey Literature Report	# not relevant
		2. QAIster	# not relevant
		3. System for information on Grey literature in Europe (OpenSIGLE)	# not relevant
		4. ProQuest Dissertation and Theses Database (PQDT)	# not relevant
		5. OpenGrey	# not relevant
		6. Grey literature report	# not relevant
5	Clinical trial	1. Clinical trial registries	# not relevant
6	Others	1. The Campbell Library (systematic review and protocol)	# for review
		2. ICL (index of chiropractic literature)	# not relevant
		3. OT seeker (occupational therapy systematic evaluation of evidence)	# not relevant
		4. PEDRO (physiotherapy evidence database)	# not relevant
		5. PILOTS (PTSD and traumatic stress)	# not relevant
		6. RehabData (rehabilitation)	# not relevant
		7. Social Care Online (social work and mental health)	# not relevant
		8. TOXNET (toxicology)	# not relevant
		9. Joanna Briggs Institute EBP Database	# not relevant
		10. TRIS (Transportation Research Information Service)	# for review
		11. IPA (International Pharmaceutical Abstracts)	# not relevant
		12. HaPI (Health and Psychosocial Instruments)	# not relevant
		13. MANTIS (Manual Alternative and Natural Therapy Index System)	# not relevant
		14. Sociological abstracts	# not relevant
		15. NAPS (new abstract and paper in sleep)	# not relevant
		16. IBIDS (International Bibliographic information on dietary supplements)	# not relevant
Total excluded		27 databases	

**Table 5:** List of eligible databases (*n* = 50).

	Database		
1	MEDLINE	26	Thai Medical Index
2	EMBASE	27	Western Pacific Region Index Medicus (WPRIM)
3	PubMed	28	WANFANG
4	Cochrane Library	29	Nikkei Asian Review
5	Cumulative Index to Nursing and Allied Health (CINAHL)	30	RISS- Korean Education and Research Information Service
6	Latin America and Caribbean Health Science Literature (LILACS)	31	Hong Kong Literature Database
7	Index Medicus for South-East Asia Region (IMSEAR)	32	Bibliography of Asian Studies
8	ScienceDirect	33	Health Research and Development Information Network (HERDIN)
9	PsycINFO	34	Cinii
10	Scopus	35	IDRnet
11	Global Health	36	EMRpub
12	Web of Science (ISI)	37	TRIP
13	Dentistry and Oral Science Source via EBSCOhost	38	ProQuest
14	Psychology and Behaviour Science via EBSCOhost	39	Science Citation Index
15	E-journal and SocINDEX and Health Business Elite via EBSCOhost	40	ERIC (Education)
16	Google Scholar	41	BNI (British Nursing Index)
17	IndMed	42	RDRB (research and development resource base- medical education)
18	KoreaMed	43	ChildData (child-related topic)
19	Chinese Biomedical Literature Database (CBM)	44	EMcare (nursing and allied health)
20	China National Knowledge Infrastructure (CNKI)	45	CommunityWISE (community issue)
21	Chinese Scientific Journal Database (VIP)	46	Global Index Medicus
22	Russian Medical and Health Journals (UDB-MED)	47	SciELO (Brazilian scientific journals)
23	SaudiMedLit	48	ComDisDome
24	Thai Index Medicus	49	Gender Watch
25	Thai Journal Citation Index Centre	50	Health and Safety Science abstract

**Table 6:** Final list of 26 databases included in the proposed systematic review.

	Categories	Databases	Notes
1.	Bibliography	MEDLINE (OVID)	
		PubMed	
		Cochrane Library	
2	Subject specific	CINAHL	
3	Regional database	KoreaMed	
		Thai Index Medicus	
		Thai Medical Index	
		IDRnet	
		EMRpub	
		Global Index Medicus	(i) WPRIM, (ii) IMSEAR, part of Global Index Medicus database
		Bibliography of Asian Studies	
		China National Knowledge Infrastructure (CNKI)	
		SaudiMedLit	
		Hong Kong Literature Database	
		Cinii	
		Health Research and Development Information Network (HERDIN)	
		LILACS	
4	Web search	Google Scholar	
5	Citation index	Scopus	
6	Others	ScienceDirect (via EBSCOhost)	
		ProQuest	(i) ComDisDome, (ii) Gender Watch, (iii) Health and Safety Science abstract (iv) BNI part of ProQuest database
		Dentistry and Oral Science Source (via EBSCOhost)	
		Psychology and Behaviour Science (via EBSCOhost)	ERIC part of EBSCOhost
		E-journal and SocINDEX and Health Business Elite (via EBSCOhost)	
		TRIP	
		Web of science	(i) SciELO, (ii) Science Citation Index part of Web of Science database

**Table 7:** Excluded databases and the reasons for their exclusion (*n*= 24).

	Name of Database	Reason for Exclusion
1	EMBASE	Not accessible via UM Library and virtual library
2	PsycINFO	Not accessible via UM Library and virtual library
3	Global health	Not accessible via UM Library and virtual library
4	IndMed	Not accessible via UM Library and virtual library
5	Chinese Biomedical Literature Database (CBM)	Not accessible via UM Library and virtual library
6	Chinese Scientific Journal Database (VIP)	Not accessible via UM Library and virtual library
7	Russian Medical and Health Journals (UDB-MED)	Not accessible via UM Library and virtual library
8	Thai Journal Citation Index Centre	Not accessible via UM Library and virtual library
9	WANFANG	Not accessible via UM Library and virtual library
10	Nikkei Asian Review	Not accessible via UM Library and virtual library
11	RISS- Korean Education and Research Information Service	Not accessible via UM Library and virtual library
12	RDRB (research and development resource base medical education)	Not accessible via UM Library and virtual library
13	ChildData (child related topic)	Not accessible via UM Library and virtual library
14	EMcare (nursing and allied health)	Not accessible via UM Library and virtual library
15	CommunityWISE (community issue)	Not accessible via UM Library and virtual library
16	ERIC	Included under EBSCOhost
17	BNI (British Nursing Index)	Included under ProQuest
18	SciELO	Include under Web of Science
19	Science Citation Index	Include under Web of Science
20	ComDisDome	Include under ProQuest
21	Gender Watch	Include under ProQuest
22	Health and Safety Science abstract	Include under ProQuest
23	Western Pacific Region Index Medicus (WPRIM)	Included under Global Medicus index
24	Index Medicus for South-East Asia Region (IMSEAR)	Included under Global index medicus
